# Barriers to COVID-19 Vaccination in a Troop of Fleet Antiterrorism Security Team Marines: Observational Study

**DOI:** 10.2196/50181

**Published:** 2024-03-19

**Authors:** E Susanne Blazek, Amy Bucher

**Affiliations:** 1 Behavioral Reinforcement Learning Lab (BReLL) Lirio Knoxville, TN United States

**Keywords:** behavioral barriers, benefits, COVID-19, Marine Corps, military, vaccine reluctance

## Abstract

**Background:**

In 2019, the World Health Organization declared the reluctance to vaccinate despite the availability of vaccination services as one of the top 10 threats to global health. In early 2021, self-reported reluctance to vaccinate among military personnel might have been considered a significant threat to national security. Having a choice architecture that made COVID-19 vaccination optional rather than required for military personnel could have inadvertently undermined military readiness if vaccination uptake did not reach an acceptable threshold.

**Objective:**

The purpose of this observational study was to examine Marines’ self-reported reasons for planning to decline the COVID-19 vaccine to understand their barriers to vaccination.

**Methods:**

As the vaccination became available to 1 company of Fleet Antiterrorism Security Team (FAST) Marines in early 2021, company command required those planning to decline vaccination to write an essay with up to 5 reasons for their choice. These essays provided the data for this study. Qualitative descriptive analysis with elements from grounded theory was used to thematically categorize FAST Marines’ written reasons for planning to decline the COVID-19 vaccine into a codebook describing 8 key behavioral determinants. Interrater agreement among 2 qualitatively trained researchers was very good (κ=0.81).

**Results:**

A troop of 47 Marines provided 235 reasons why they planned to decline the COVID-19 vaccine. The most frequent reasons were difficulty understanding health information (105/235, 45%), low estimates of risk (33/235, 14%), and fear of physical discomfort (29/235, 12%). Resulting interventions directly targeted Marines’ self-reported reasons by reducing barriers (eg, normalized getting the vaccine), increasing vaccine benefits (eg, improved access to base gyms and recreational facilities), and increasing nonvaccine friction (eg, required in writing 5 reasons for declining the vaccine).

**Conclusions:**

Understanding the barriers military personnel experience toward COVID-19 vaccination remains critical as vaccine acquisition and availability continue to protect military personnel. Insights from subpopulations like FAST Marines can enhance our ability to identify barriers and appropriate intervention techniques to influence COVID-19 vaccination behaviors.

## Introduction

In 2019, the World Health Organization declared the reluctance to vaccinate despite the availability of vaccination services as one of the top 10 threats to global health [1]. Since COVID-19 emerged in late 2019, COVID-19 vaccination specifically has been deemed a critical public health priority [2]. Vaccination is associated with reduced hospitalization and death rates following COVID-19 infection [3-5], as well as reductions in transmission and severity of infection [5,6]. Yet, it has proven challenging to achieve the desired rates of vaccination in the United States generally [7] and within specific subpopulations such as rural dwellers [8], political conservatives [9], and people with previous diagnoses of COVID-19 [10].

Combating uneven and inadequate uptake of COVID-19 vaccines requires an understanding of the behavioral determinants (ie, barriers and facilitators of the behavior) leading people to vaccinate or not [11]. Behavioral design frameworks, such as the COM-B (capability, opportunity, motivation, behavior) model of behavior change which suggests that capability, opportunity, and motivation are essential for behavior change, provide a categorization scheme for behavioral determinants that then allows interventionists to select techniques that target the specific barriers preventing people from completing a desired behavior [12-16]. While there are some behavioral determinants that may be addressable through directives from credible sources, as traditional public health campaigns may be described, these campaigns are unlikely to be broadly successful or sufficient in isolation to promote widespread behavior change. This is because they do not adequately account for the breadth of people’s barriers to performing the behavior or adequately enhance the benefits of the behavior [17,18]. Counteracting a crisis like the COVID-19 pandemic necessitates understanding how people make decisions, what gets in the way, and what gets people to take action [19]. Qualitative research approaches that yield richer insights might contribute to a better understanding of these nuances [20-23].

Barriers to COVID-19 vaccination in the general population include, among others, beliefs of low susceptibility to infection, perceived low severity of COVID-19, beliefs of low vaccine effectiveness, concerns about side effects, desires for more information, and not having explicit endorsement from a health care provider [24,25]. Not having received a vaccine in the past year is also associated with lower vaccination rates [26]. Barriers differ for population subgroups based on characteristics such as geographical location, work responsibilities, income level, educational attainment, and insurance status [25], several of which are social determinants of health. To effectively influence vaccination behavior within a population subgroup, researchers should seek to understand the specific barriers prevalent in that group.

One such group is military personnel and veterans. A specific study of the behavioral determinants of COVID-19 vaccination in military personnel is critical, as vaccination against infectious diseases supports operational readiness [27]. Moreover, given military veterans’ high rates of mental health issues and suicide, there has been some effort to consider veteran status as a social determinant of health [28]. A better understanding of the health decisions of military personnel therefore has the added potential of supporting improved health equity.

In early 2021, medical experts and government leaders considered the lack of COVID-19 vaccination among military personnel a potential threat to national security [29,30]. Unlike influenza, tetanus, diphtheria, pertussis, smallpox, and other vaccines, which are required aspects of force readiness, the COVID-19 vaccine was initially optional for military personnel before becoming mandatory [31] and finally becoming optional again on January 10, 2023 [32]. Having a choice architecture that makes COVID-19 vaccination optional for military personnel [33] could inadvertently undermine military readiness if vaccination uptake does not reach an acceptable threshold. Therefore, it is important to understand the reasons why military personnel might not vaccinate to appropriately intervene on those barriers.

In the United States, military personnel are socialized to a specific set of values, including honor, bravery, and personal sacrifice [34]. Each branch of the US military then has characteristics that may influence the specific behavioral determinants of its troops toward vaccination. For example, within the United States Marine Corps, values such as the need for control and independence are reinforced, sometimes to the detriment of health-promoting behaviors [35,36]. Marines report a lower likelihood of vaccine receipt than other branches of service [37,38]. These differences may reflect true differences between the branches or, more likely, may reflect underlying differences in the characteristics of individuals choosing to serve in different branches.

Junior Marines (E1-E4) experiencing a high degree of external control and loss of agency due to service [39] might feel an enhanced need to establish autonomy when given the chance, such as by declining an optional vaccine. Research has found that military personnel ranked E1-E4 were the most likely to reject an avian influenza vaccine [37]. While the Marine Corps has embraced an approach of informed health care choice for its members, there is a rocky history of medical paternalism (eg, anthrax vaccination in the 1991 Gulf War, but see [40]) that may negatively influence modern Marines’ perceptions of vaccination campaigns.

Given their overall health and fitness and the risks that come with their jobs, Marines may also have a low perceived personal risk from the COVID-19 infection and a tolerance for risk that others may find uncomfortably high. Although data were limited at the time, it was projected in early 2021 that those who contracted COVID-19 had a 0.5%-5% chance of developing a critical illness requiring intensive care or resulting in death [41]. Those odds might have seemed acceptable to Fleet Antiterrorism Security Team (FAST) Marines, who are trained to provide security forces to guard high-value naval installations, most notably those containing nuclear vessels and weapons. FAST Marines find themselves in high-risk situations as part of their regular training, for example with live fire, extreme exposure to the elements, and sleep and nutrition deprivation. FAST Marines’ daily work likely undermines their perceived severity of contracting COVID-19. In contrast, the perceived severity of contracting anthrax among 22 Department of Defense (DOD) personnel in 2015 resulted in near 100% adherence to a complex emergency postexposure prophylaxis following accidental exposure to live *Bacillus anthracis* spores [42].

Americans’ willingness to get the COVID-19 vaccine declined from 72% in May 2020 to 51% in September 2020 [43] and rebounded to 60% in November 2020 [44]. Surveys from early 2021 found that fewer than half of military troops and military spouses planned to receive the vaccine [45]. At the time of the study, FAST Marines in Yorktown, Virginia, were in a privileged position: they were 1 of 12 bases nationwide to be offered the COVID-19 vaccine before widespread availability [46]. Yet an informal poll of Marines in 1 FAST company showed that only 10% planned to get the COVID-19 vaccine, much lower than other estimates at the time [43,44]. The FAST Marines’ company command introduced key friction to those FAST Marines who planned to decline the COVID-19 vaccine: they had to write formal papers outlining up to 5 reasons why. This observational study makes use of those written data to understand FAST Marines’ barriers to COVID-19 vaccination.

## Methods

### Ethical Considerations

We followed the Standards for Reporting Qualitative Research [47]. This research was approved by an Exception Determination Official with the Naval Medical Center Portsmouth Research Subjects Protection Division. The study does not meet the Naval Medical Center Portsmouth Research Subjects Protection Division’s definition of research in accordance with 32 CFR 219.102 and DoDI 3216.02. Therefore, no written consent was obtained for the qualitative analyses. Study data were deidentified.

### Sample

The FAST platoon is the core operational unit of the Marine Corps Security Force Battalion. There are 18 FAST platoons divided into 3 FAST companies that are based in Norfolk and Yorktown, Virginia. The battalion commander of 1 of the 3 FAST companies asked his Marines to either obtain the COVID-19 vaccine or to provide, in writing, at their own leisure but with a deadline, up to 5 reasons why they did not want the COVID-19 vaccine. One platoon of 47 male junior FAST Marines (ie, E1-E4) exclusively chose to provide written reasons for declining the COVID-19 vaccine. Their responses were collected by the battalion commander, entered into Microsoft Excel (Microsoft Corp) without demographic information, and shared with the researchers. Demographic data were not collected, and Marines did not receive compensation because the writing task was originally intended as a troop exercise and an intervention to promote COVID-19 vaccination, not a research project. Data were collected in February 2021. There were no efforts to retroactively identify which FAST Marine submitted which reasons or determine their demographics or ranks.

### Qualitative Descriptive Analysis

The purpose of our study, to examine FAST Marines’ self-reported reasons for planning to decline the COVID-19 vaccine to understand their barriers to vaccination, guided our choice of qualitative descriptive analysis. Qualitative description is an appropriate approach to gathering insights on novel or poorly understood phenomena [20]. We conducted a rapid review to develop a codebook of empirically supported barriers to obtaining a COVID-19 vaccine that we coded the FAST Marines’ responses against. We used thematic analysis [48,49] with elements from grounded theory [50,51]. The 2 qualitatively trained researchers independently coded the responses in Excel. We calculated interrater agreement with Cohen’s κ [52], which accounts for change agreements. The interrater agreement was very good (κ=0.81). See Table 1 for the final codebook consisting of 8 codes.

**Table 1:** The codebook used for qualitative descriptive analyses of 47 Yorktown, Virginia, Fleet Antiterrorism Security Team Marines’ 235 written reasons for why, in February 2021, they planned to decline the COVID-19 vaccine.

Label	Definition	Description of how to know when code occurs	Keywords or phrases	Positive example (is this code)	Negative example (is not this code)
Difficulty understanding health information [53,54]	Marines do not understand or misunderstand basic medical information.	Responses that inadequately represent (most likely due to honest lack of understanding) what is currently known about the vaccine’s ingredients, effectiveness, side effects, outcomes, etc.	FDA^a^; CDC^b^; mRNA^c^; effective; effectiveness; study; studies; too soon; early to know; immunity; side effects; takes years; not approved; not proven	“I’ve heard that [the vaccine] makes you very susceptible to sickness and weakens your immune system.” “MRNA vaccines have never been used before”	“It is my right to refuse it” “It uses fetus and fetus cell lines of aborted babies”
Perceived low susceptibility of COVID-19 and its severity [55]	Marines underestimate their risk of contracting COVID-19 and of a serious negative outcome.	Responses that suggest a low expected likelihood of contracting COVID-19 and of falling seriously ill.	No symptoms; young; healthy; likelihood; slim; survival rate; no danger	“I don’t have COVID and have never had a single symptom of it, so therefore I don’t need it.” “Flu hasn’t affected me any different”	“Can cause you to be sterile” “I don’t like needles”
Concern over vaccine safety and potential side effects [56]	Marines expect the vaccine or side effects will cause physical pain or discomfort.	Responses mentioning known and unpleasant side effects.	Needles; side effects; symptoms; allergic reaction; getting sick	“I don’t like needles” “Possibly getting sick and being unable to train in DM course”	“Not FDA approved/licensed” “I don’t want it”
Status quo bias may impact preventive pandemic behaviors [56,57]	Marines postpone getting the vaccine if doing so does not have an immediate benefit for them.	Responses mentioning drawbacks to getting vaccinated and no benefits.	No benefit; not a requirement; voluntary; wear mask; socially distance	“Not a requirement for the Marine Corps or deployment.” “There isn’t any benefit to getting the vaccine such as being immune to COVID-19 and not having to wear a mask or maintain 6 feet of social distance”	“I’ve already received a flu shot” “I have other things to worry about”
Distrust in health care [58]	Marines distrust the health care system in general or have had bad experiences in health care settings.	Responses mentioning trust in the health care system, in the CDC, and in the vaccine.	Trust; Pfizer; questionable history; rushed; wrong about the virus	“I don’t trust it at all” “I rarely trust anything that is newly developed with no history of defects or flaws.”	“I’d rather wait and see how people’s body respond to it” “Standard set low, CDC said it only needed to be 50% effective”
Social norms may influence attitudes and beliefs about vaccines [55,59]	Marines have no role models—family members, community members, etc—who are seen endorsing the behavior.	Responses indicating that trusted friends, family, and health care providers are not getting vaccinated.	Family; peers; Marines; doctor; friends	“Other marines I trust aren’t getting it” “I asked my doctor from home & he said wait a couple years”	“Side effects are negative” “Short term effects show facial paralysis in several cases”
Believing in COVID-19 conspiracies [60]	Marines have strong, irrational, and disproven beliefs against the vaccine.	Responses that indicate believing in nonvalidated, mis- or disinformation about the COVID-19 vaccine.	Test dummy; guinea pig; political gain; election time; media; democrats	“Don’t want to be a test dummy for the military.” “Can cause you to be sterile.”	“The vaccine is not guaranteed to work” “Flu has been around for 500 years and finally got vaccinated in 1930 by injecting a weaker virus into you”
Other or unclear	Combination of categories with fewer than 1% of responses each.	Responses with prosocial beliefs, religious concerns, or vague.	Does not fit in any other category.	“It goes against my religious beliefs” “Personal beliefs”	“99.98% survival rate for my age group” “I simply don’t want the vaccine... Or is it mandatory?”

^a^FDA: US Food and Drug Administration.

^b^CDC: Centers for Disease Control and Prevention.

^c^mRNA: messenger RNA.

## Results

Frequencies of coded responses can be found in Table 2. FAST Marines’ reasons for planning to decline the COVID-19 vaccine were categorized into the following 7 barriers: difficulty understanding health information (105/235, 44.7%), perceived low susceptibility of COVID-19 and its severity (33/235, 14%), concern over vaccine safety and potential side effects (29/235, 12.3%), status quo bias may impact preventive pandemic behaviors (28/235, 11.9%), distrust in health care (12/235, 5.1%), social norms may influence attitudes and beliefs about vaccines (12/235, 5.1%), and believing in COVID-19 conspiracy theories (5/235, 2.1%). The last 4.7% (11/235) of responses were categorized as other or unclear.

**Table 2:** Summary of the behavioral determinants of COVID-19 vaccination uncovered by qualitative descriptive analyses of 47 Yorktown, Virginia, Fleet Antiterrorism Security Team Marines’ 235 written reasons for why, in February 2021, they planned to decline the COVID-19 vaccine.

Determinant	Values (n=235), n (%)
Difficulty understanding health information	105 (44.7)
Perceived low susceptibility to COVID-19 and its severity	33 (14)
Concern over vaccine safety and potential side effects	29 (12.3)
Status quo bias may impact preventive pandemic behaviors	28 (11.9)
Distrust in health care	12 (5.1)
Social norms may influence attitudes and beliefs about vaccines	12 (5.1)
Believing in COVID-19 conspiracy theories	5 (2.1)
Other or unclear	11 (4.7)

A total of 45% (105/235) of all responses (each FAST Marine gave up to 5), and 3 times as many as the second largest barrier, had to do with difficulty understanding medical information. In fact, 96% (45/47) of FAST Marines—all except 2 FAST Marines—indicated difficulty understanding medical information, ranging from the inability to understand basic medical information to the inability to grasp nuances in the medical literature.

## Discussion

### Overview

A qualitative descriptive analysis of 235 reasons for planning to decline the COVID-19 vaccine provided a contemporaneous understanding of the barriers to vaccination experienced by an infrequently studied population subgroup: junior FAST Marines. FAST Marines’ barriers to COVID-19 vaccination included low health literacy, perceived low susceptibility to COVID-19 and its severity, concern over vaccine safety and potential side effects, and other less frequently reported barriers. These findings are consistent with work on Air Force personnel who self-identified as vaccine-hesitant and indicated concerns about efficacy, side effects, and vaccine-induced illness as top barriers to vaccination [61], while adding the insight that low health literacy may be associated with such concerns.

Our findings extend previous work on barriers to COVID-19 vaccination in part because we captured in-the-moment attitudes, beliefs, and cognitions about the COVID-19 vaccine in a less frequently studied population subgroup: junior FAST Marines. The barriers described in the written responses were not subject to the same demand characteristics as barriers elicited through formal research. That is, FAST Marines were not restricted to a preselected list of barriers to COVID-19 vaccination as might have been the case in survey research, nor were they aware of a research framing that might have prompted more socially acceptable responses.

### Impact on Future Interventions Suggested by Findings

Fear is a common, if not primary, motivator used by Marine Corps leadership. Instead of fear-based messaging, which might induce reactance [62,63], and based on our results from the qualitative descriptive analysis, we recommended to the company command placing emphasis on the fact that *because* FAST Marines are young and healthy, they must get the COVID-19 vaccine. This kind of messaging emphasizes resilience and self-efficacy [55,63] and builds on FAST Marines’ esprit de corps. Future research should evaluate building upon the positive and strong military identity [34] to promote health behaviors in military personnel.

What makes Marines great at their jobs might get in the way of success elsewhere. That is, feeling invincible could positively support junior Marines in performing their job but could become a barrier when they decide whether or not to get the COVID-19 vaccine. Our results do not suggest or endorse changing traits or skills that make Marines great at their jobs. Instead, the results suggest using that information to better speak the Marines’ language and motivate them to get vaccinated. In this case, intervention design should not treat some of these qualities as barriers to overcome so much as consider them to be facilitators to be leveraged.

The study results provided insights for behavioral intervention at the troop and company levels. Company command developed interventions that directly targeted FAST Marines’ self-reported reasons by increasing nonvaccine friction (eg, required in writing 5 reasons for declining the vaccine), reducing barriers (eg, normalized getting the vaccine by encouraging senior personnel to get vaccinated), and increasing vaccine benefits (eg, provided fringe benefits to vaccinated FAST Marines such as the higher likelihood of approval for out-of-area leave).

### Limitations and Directions for Future Research

The primary limitation stems from the nature of the data. The 235 written reasons to decline the COVID-19 vaccine were intended as a troop exercise and not a scientific study. We were unable to explore demographics known to be determinants of COVID-19 vaccination because of the deidentified data.

The second limitation involves our inability to determine our results’ direct impact on COVID-19 vaccination in the troop of FAST Marines. Company command introduced a number of behavioral interventions following this study but implemented them inconsistently across the troops within the company. The goal of managing COVID-19 transmission superseded scientific rigor. It is therefore difficult to determine why only 10% (11/114) of FAST Marines in the company expressed intentions to accept the COVID-19 vaccine in January of 2021 but, 8 weeks later, 84% (105/114) of the company had chosen to receive the vaccine. Some FAST Marines likely chose to vaccinate due to the interventions implemented by their leadership to address their barriers, but it is also likely that the intention-behavior gap accounts for some of the discrepancy [23]. Future research on vaccine campaigns in the military should explore the impact of behavioral interventions like the ones implemented here and should also capture how vaccines are administered: with informed choice, under time pressure, etc (see Murphy et al [40]).

### Conclusion

Understanding the barriers military personnel experience toward COVID-19 vaccination remains critical despite the success of COVID-19 vaccination to date, with 96% of US military personnel fully vaccinated as of January 2023 [32], compared to roughly 70% for all Americans [64]. It is hard to overstate the impact of even small wins when it comes to the health of military personnel. Vaccine acquisition and availability continue protecting military personnel, and the DOD currently offers 17 different vaccines for the prevention of infectious diseases among military personnel, where appropriate [65]. The historical and cultural context of the Marine Corps, and specifically the Marine Corps’ FAST, likely gave rise to a particular constellation of barriers to the COVID-19 vaccine. Identifying these barriers early and as the COVID-19 vaccine was made available helped shape how a FAST company command successfully ensured vaccine participation. With the return to a choice architecture that makes ongoing COVID-19 vaccination voluntary, coupled with the knowledge that ongoing boosters will be necessary to maintain resistance to infection, military leadership has ample opportunity to continue to offer behavioral interventions tailored to their troops’ needs.

## Abbreviations

COM-B: capability, opportunity, motivation, behavior
DOD: Department of Defense
FAST: Fleet Antiterrorism Security Team

## Data Availability

The data sets generated and analyzed during this study are not publicly available due to a lack of ethics approval to share.
